# *Actinobacillus pleuropneumoniae* Surviving on Environmental Multi-Species Biofilms in Swine Farms

**DOI:** 10.3389/fvets.2021.722683

**Published:** 2021-09-30

**Authors:** Abraham Loera-Muro, Flor Y. Ramírez-Castillo, Adriana C. Moreno-Flores, Eduardo M. Martin, Francisco J. Avelar-González, Alma L. Guerrero-Barrera

**Affiliations:** ^1^CONACYT-Centro de Investigaciones Biológicas del Noreste, La Paz, Mexico; ^2^Laboratorio de Biología Celular y Tisular, Departamento de Morfología, Centro de Ciencias Básicas, Universidad Autónoma de Aguascalientes, Aguascalientes, Mexico; ^3^Laboratorio de Estudios Ambientales, Departamento Fisiología y Farmacología, Centro de Ciencias Básicas, Universidad Autónoma de Aguascalientes, Aguascalientes, Mexico

**Keywords:** *Actinobacillus pleuropneumoniae* (APP), biofilms, drinking water, swine farms, environmental multi-species biofilms

## Abstract

*Actinobacillus pleuropneumoniae* is the etiologic agent of porcine contagious pleuropneumonia, an important respiratory disease for the pig industry. *A. pleuropneumoniae* has traditionally been considered an obligate pig pathogen. However, its presence in the environment is starting to be known. Here, we report the *A. pleuropneumoniae* surviving in biofilms in samples of drinking water of swine farms from Mexico. Fourteen farms were studied. Twenty drinking water samples were positive to *A. pleuropneumoniae* distributed on three different farms. The bacteria in the drinking water samples showed the ability to form biofilms *in vitro*. Likewise, *A. pleuropneumoniae* biofilm formation *in situ* was observed on farm drinkers, where the biofilm formation was in the presence of other bacteria such as *Escherichia coli, Stenotrophomonas maltophilia*, and *Acinetobacter schindleri*. Our data suggest that *A. pleuropneumoniae* can inhabit aquatic environments using multi-species biofilms as a strategy to survive outside of their host.

## Introduction

*Actinobacillus pleuropneumoniae*, a member of the *Pastereullaceae* family, is a Gram-negative, mobile, and rod-shaped bacterial pathogen. *A. pleuropneumoniae* is the etiologic agent of porcine contagious pleuropneumonia that causes great economic losses in the pig industry (1, 2). *A. pleuropneumoniae* resides in the upper respiratory tract in subclinically infected or colonized pigs, and transmission from pig to pig occurs mainly by direct oral, nasal contact, or by droplets of aerosol spread over short distances (2, 3). Many virulence factors have been reported in *A. pleuropneumoniae* including lipopolysaccharide (LPS), exotoxins (Apx), polysaccharide capsule, protease (4), urease, iron acquisition proteins, and enzymes involved in anaerobic respiration, which may contribute to the disease (5, 6). Likewise, some putative adhesion type IV pilus (7), Flp pilus (8), autotransporter adhesins (9), and biofilm formation have been observed (10). Among these, Apx toxins are the major virulence factors involved in the pathogenesis of pleuropneumonia (2).

Nineteen serovars of *A. pleuropneumoniae* have been proposed based on capsular antigens (11–13). Two biovars have been described based on nicotinamide adenosine dinucleotide (NAD) requirements. Serovars 1–12 and 15–16 usually belong to biovar I, which contains NAD-dependent strains and is commonly implicated in pneumonic processes. Serovars 13 and 14 are usually NAD-independent and belong to biovar II (2). Serovars are obligate pathogens but differ in virulence traits and regional distribution. Serovars 1, 5, and 7 are predominant in North America; serovar 2 is most common in Europe; and serovars 1, 3, 4, 5, and 7 are typically isolated in China (14). Atypical *A. pleuropneumoniae* serovar 13 strains have been detected in North America, Canada, and United States (US). However, these strains are NAD-dependent (biovar I), and antigenically and genotypically different from the European strains, including the reference strain (15). In Mexico, serovars 1, 3, 5, and 7, which belong to biovar I, are generally found. Subtype 1a from serovar 1 has been associated with acute cases of the disease in this country (16).

Biofilms are communities composed of bacterial cells embedded in a matrix of polymers that are adhered to a surface. These structures help bacteria to survive in hostile environments for their development, such as desiccation or nutrient starvation (17, 18). Biofilms constitute the prevalent survival strategy for microorganisms in the environments (19–21). Moreover, multi-species biofilms represent the most important lifestyles of microorganisms in nature (21). Interspecies interactions can drive ecological advantages in a biofilm. Multi-species interactions are also involved in the persistence of pathogens on inert surfaces (20). Tremblay et al. (22) found that *A. pleuropneumoniae* can form biofilms in infected pigs, demonstrating for the first time that *A. pleuropneumoniae* biofilm occurs during the infection process. Our group and, recently, an independent group from China both demonstrated that *A. pleuropneumoniae* can form multi-species biofilms in combination with other porcine respiratory pathogens, as well as with other bacteria such as *Escherichia coli* and *Staphylococcus aureus* under laboratory conditions (23–25). Additionally, our group reported the presence of *A. pleuropneumoniae* in drinking water from pig farms in Mexico using a specific PCR for the RTX toxin gene *apxIV* (26, 27). *A. pleuropneumoniae* has traditionally been considered an obligate pig pathogen. However, there are not many reports about its presence in the environment. The presence of *A. pleuropneumoniae* in farm drinking water was confirmed by indirect immunofluorescence using an *A. pleuropneumoniae*-specific polyclonal antibody and by fluorescent *in situ* hybridization (FISH) (26). In this work, the presence of *A. pleuropneumoniae* in multi-species biofilms in samples of swine drinking water, and in environmental multi-species biofilms formed in drinkers in swine farms is reported.

## Materials and Methods

### Sampling Procedures

For this study, a resampling of 14 swine farms, some previously sampled in Loera-Muro et al. (26) were done. A total of 84 samples of drinking water were obtained. The sampling period was from June 2011 to February 2012, and the experimental analysis of 2011–2014. All farms are located within the State of Aguascalientes, Mexico. This area is characterized by a semiarid zone with little rain in summer, very short winters, and temperatures rarely dropping at 4°C, with the remainder of the year with a warm to temperate climate (20°C), mostly sunny with temperatures above 30°C. The farms are family farms without technology, where animals are in pens covered with a roof. The farms had an average population of 100 pigs in a fattening stage. The farms included in the study presented both systems of drinkers: watering places and nipple drinkers for the animals. Water samples were taken from drinkers randomly. Water samples were taken in two different ways: (i) a set of samples was collected directly from the watering places (show Supplementary Material S1) in the deeper zone with sterile Corning tubes of 50 ml far to animals, and (ii) the other set of samples was taken from the taps that were previously sterilized at the tip of the tap from the nipple drinker with 70% ethanol and lighter for 60 s, and then the water was left to run 60 s before sampling (26). Water samples were stored at room temperature for 7 days until used.

### DNA Extraction

For water samples, first, the samples were shaken for approximately 30 s. Then, 3 ml of each sample was taken and centrifuged at 10,000 × g (Universal 320R, Hettich) for 10 min. The supernatant was discarded, and the pellet was used for DNA extraction. DNA extraction from water samples was performed as described by Loera-Muro et al. (26) and stored at −20°C until use. Positive controls for this study were *A. pleuropneumoniae* serovar 1-4074, 3, 4, and 10, and *E. coli* ATCC 25922. *A. pleuropneumoniae* control strains were kindly provided by Dr. Mario Jacques, from Université de Montréal. *A. pleuropneumoniae* strains were grown on brain heart infusion agar plates (BHI; Bioxon, Mexico) supplemented with 15 μg NAD ml^−1^. *E. coli* ATCC 25922 was grown on BHI. All the bacterial strains were incubated at 37°C overnight, and then DNA extractions were performed as described above.

### PCR to Detect *apx* Toxin Genes and 16S rDNA

For detection of the *apxIV* gene, specific for *A. pleuropneumoniae*, the assay was performed as described by Frey et al. (28) in a final volume of 25 ml (26). For the detection of *apxIA, apxIB, apxII*, and *apxIII* genes coding for the Apx toxins of *A. pleuropneumoniae*, the methodology described by Rayamajhi et al. (29) was used with modifications. The PCR conditions were as follows: 94°C for 5 min followed by 30 cycles of 30 s at 94°C, 60 s at 69°C for *apxIB* and *apxIII*, 66°C for *apxII*, and 72°C for *apxIA*, and 3 min at 72°C with a final elongation step at 72°C for 10 min. For the *apxIV* gene, PCR conditions were as follows: 95°C for 1 min followed by 30 cycles of 94°C for 30 s, 54°C for 30 s, and 72°C for 1 min with a final elongation step at 72°C for 5 min. The primer sequences are shown in Table 1. The amplification products were observed by electrophoresis in 1.5% agarose gel stained with 1 μg ml^−1^ ethidium bromide. Images of gels were captured using the Chemi Doc (BioRad), image analyzer, and the software Quantity One (Bio-Rad, California, USA). PCR products were purified with the QIAquick PCR purification kit (Qiagen, Valencia, CA). The concentration of amplicons was estimated spectrophotometrically with a Nanodrop NanoPhotometer^TM^ Pearl (Implen GmbH, Germany). Sequencing of the PCR products was carried out at the LANBAMA (Laboratorio Nacional de Biología Agrícola, Médica y Ambiental, IPICYT, Mexico) with the same primers used for detection of the *apx* genes. Sequences were compared to the databases at GenBank and with the program Jalview 2.9. PCR detection of 16S rDNA and sequencing from drinking water samples were made at the Molecular Biology Diagnostic Laboratory of Veterinary Medicine Faculty of Université de Montréal.

**Table 1:** Primers sequences used in this study.

**Name**	**Sequence (5^′^-3^′^)**	**Product size (bp)**	**References**
APXIVANEST1L	GGG GAC GTA ACT CGG TGA TT	377	Frey et al. (28)
APXIVANEST1R	GCT CAC CAA CGT TTG CTC		
ApxIAF	ATCGAAGTACATCGCTCGGA	723	Rayamajhi et al. (29)
ApxIAR	CGCTAATGCTACGACCGAAC		
ApxIBF	TTATCGCACTACCGGCACTT	811	Rayamajhi et al. (29)
ApxIBR	TGCAGTCACCGATTCCACTA		
ApxIIF	GAAGTATGGCGAGAAGAACG	965	Rayamajhi et al. (29)
ApxIIR	CGTAACACCAGCAACGATTA		
ApxIIIF	GCAATCAGTCCATTGGCGTT	396	Rayamajhi et al. (29)
ApxIIIR	GACGAGCATCATAGCCATTC		

### Biofilm Formation Assay

Two trials to observe biofilm formation in the water samples were performed. First, water samples were screened using the glass tube biofilm assay as described previously by Jin et al. (30) with modifications. The water samples were shaken during 30 s approximately. Then, 2 ml of water samples were taken, and mixed with 20 ml of BHI broth (1/10). The samples were poured in a petri dish with a sterile slide and incubated overnight at 37°C. The second test was performed directly at the swine farm. This experiment was done at the swine farm belonging to the Center of Agricultural Sciences at the Universidad Autónoma de Aguascalientes, where *A. pleuropneumoniae* was previously detected in samples of drinking water. For this experiment, a portable device was designed that allows biofilm formation on flat surfaces such as slides, and which can be positioned at different levels of depth on the drinkers (Supplementary Figure 1) (Patent in process: No. MX/E/2014/054592). This portable device was sterilized by autoclaving and then it was placed in the drinkers of the farm taking care that the water completely covered them. Periodically, we checked that the portable device remained within the drinkers and that they were not turned. The experiment was carried out for 7 days. After that time, the portable devices were carefully removed and stored in a cooler immediately. Once at the laboratory, the slides were removed and dried at 37°C for 30 min. FISH assays were performed on all samples.

### Fluorescent *in situ* Hybridization Assay

The FISH assay was performed as described previously Loera-Muro et al. (23) with modifications. *A. pleuropneumoniae* serovar 1-4074 was used as a positive control, and *E. coli* ATCC 25922 was used as a specific control. The samples were treated with 80% ethanol for 20 min to fix the sample. Then, the slides were subjected to pretreatment with sodium citrate (1 mM) at 95°C for 5 min. Samples were washed with distilled water at 50°C for 5 min, removed, and dried in an incubator at 37°C. Aliquots (30 μl) containing the following hybridization mixture were applied to each slide: 10 mM NaCl (J.T. Baker, Xalostoc, Mexico), 50 mM Tris-HCl (Invitrogen, Carlsbad, California, USA) (pH 7.5), 10% (w/v) sodium dodecyl sulfate (Sigma, Steinheim, Germany), 30% (v/v) formamide (Pharmacia Biotech AB, Uppsala, Sweden), 0.1% (v/v) Triton X-100 (USB, OH, USA), and a fluorescent probe with a final concentration of 1 μM. The probes APXIVAN L (GGG GAC GTA ACT CGG TGA TT) and APXIVAN R (GCT CAC CAA CGT TTG CTC) were labeled with fluorescein (FITC) or tetramethylrhodamine (TAMRA) in the N-terminus (5′) (Alpha DNA Montréal, Canada). The slides were covered with cover glass and then placed in a preheated moisture chamber in the dark at 55°C overnight. After this, slides were washed in preheated washing buffer (5 mM Tris, 15 mM NaCl, and 0.1% [v/v] Triton X-100 [pH 10]) at a hybridization temperature for 30 min. Following a brief immersion in bi-distilled water, slides were air-dried and mounted with one drop of ProLong Gold Antifade Mountant (Invitrogen Oregon, USA) with or without 4′-6-Diamidino-2-phenylindole (DAPI) (Invitrogen Oregon, USA). The samples were stored at −20°C in the dark until laser scanning confocal microscopy (LSCM) (Leica, Germany) or epifluorescence microscope (EM) (Leica, Germany) observation.

### Biofilm Analysis by Scanning Electron Microscopy

Positive samples of *A. pleuropneumoniae* for biofilm formation were analyzed by scanning electron microscopy (SEM). The samples were processed as described by Baum et al. (31) with modifications by Loera-Muro et al. (26). Samples were observed with a Jeol LV-5900 scanning electron microscope.

## Results

### Detection of *apx* Genes

**Figure 1:**
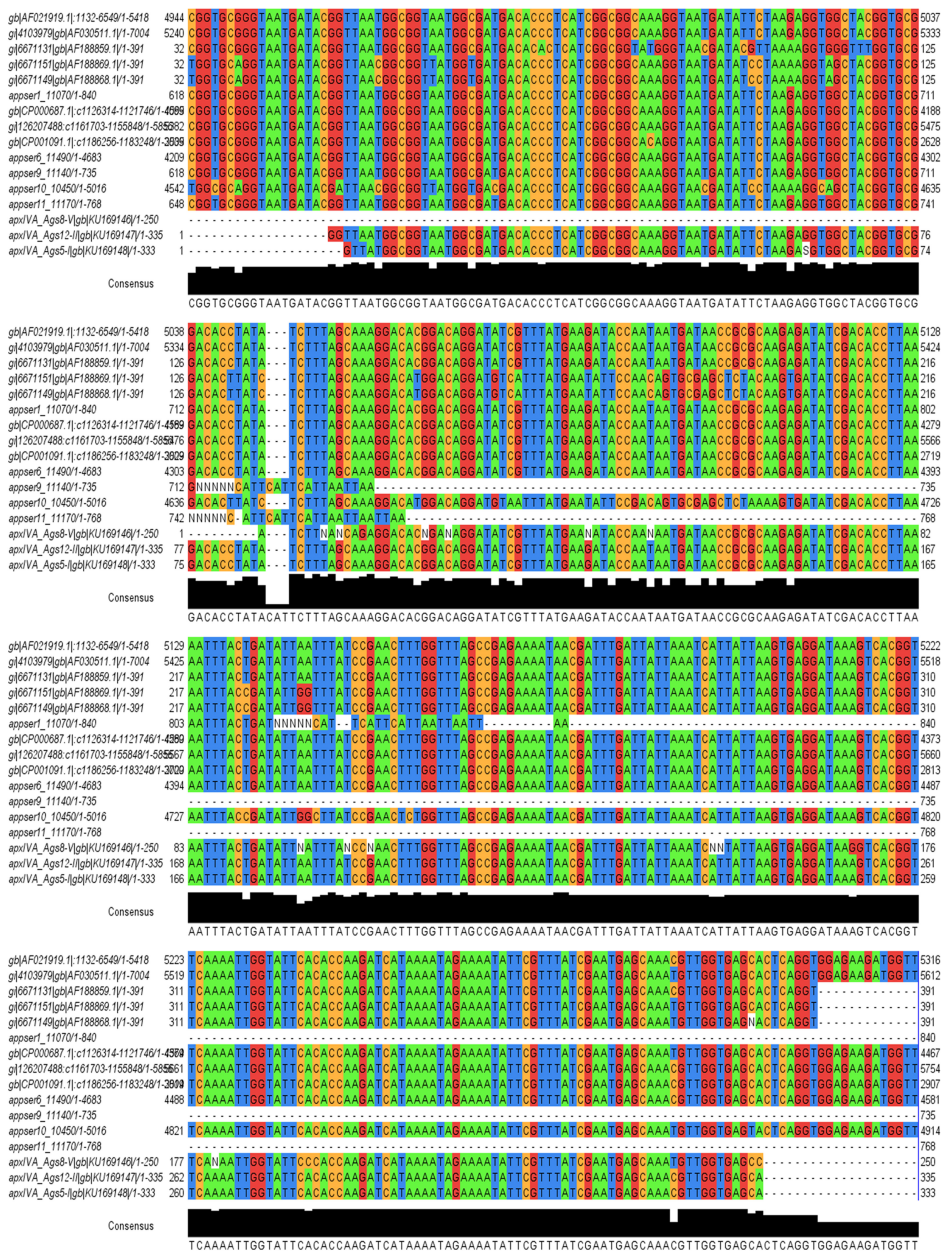
Alignment of sequences with the program Jalview 2.9 of *A. pleuropneumoniae* specific gene *apxIV* obtained from samples of drinking water from pig farms. Samples randomly selected and positive for alignment with the sequences deposited in GenBank of *apxIV* gene are ApxIVA_Ags5-I (ApxIVA_Ags5-I/gb/KU169148/1-333), ApxIVA_Ags8-V (ApxIVA_Ags8-V/gb/KU169146/1-250), and ApxIVA_Ags12-II (ApxIVA_Ags12-II/gb/KU169147/1-335).

Total DNA was extracted from samples of drinking water to search for the pathogen *A. pleuropneumoniae* by PCR. The assay was based on the detection of the *apxIV* toxin gene of *A. pleuropneumoniae*, which is specific for this pathogen. The amplicons generated were analyzed by agarose gel electrophoresis, and their size was calculated by comparison with a molecular weight marker. The product was 377 bp. from 84 samples of drinking water, 20 were positive by PCR (Table 2 and Supplementary Figure 2). The samples were coming from three different farms (Table 2). Of the 20 positive samples, three amplicons were taken randomly for sequencing: (i) ApxIVA_Ags5-I from Ags5-I, (ii) ApxIVA_Ags8-V from Ags8-V, and (iii) ApxIVA_Ags12-II from Ags12-II. All the sequences obtained were specific for the *A. pleuropneumoniae apxIV* gene (Figure 1). The sequences were uploaded to GenBank (GenBank accession numbers: KU169148 [ApxIVA_Ags5-I], KU169146 [ApxIVA_Ags8-V], and KU169147 [ApxIVA_Ags12-II]). However, only three samples were positive for the *apxIB* gene (Ags5-I, Ags5-II, and Ags5-III), two samples were positive for the *apxII* gene (Ags5-II and Ags8-VI), and any sample was positive for *apxIA* and *apxIII* genes (Table 2; Supplementary Figure 2). Those data suggest that *A. pleuropneumoniae* serovar 7 was present in the drinking water samples of Aguascalientes farm.

**Table 2:** Properties of 20 positive samples of *A. pleuropneumoniae* from drinking water of swine farms in Mexico.

**No**.	**Sam-ple name**	***apxIA***	***apxIB***	***apxII***	***apxIII***	***apxIV***	***apxIV*** **FISH**	**Biofilm formation**
(1)	Ags5-I*	–	+	–	–	+	+	††††
(2)	Ags5-II	–	+	+	–	+	+	††††
(3)	Ags5-III	–	+	–	–	+	+	††††
(4)	Ags5-IV	–	–	–	–	+	+	†
(5)	Ags5-V	–	–	–	–	+	+	†
(6)	Ags5-VI	–	–	–	–	+	+	†
(7)	Ags8-I	–	–	–	–	+	+	†
(8)	Ags8-II	–	–	–	–	+	+	†
(9)	Ags8-III	–	–	–	–	+	+	†
(10)	Ags8-IV	–	–	–	–	+	+	†
(11)	Ags8-V*	–	–	–	–	+	+	†
(12)	Ags8-VI	–	–	+	–	+	+	†
(13)	Ags12-II*	–	–	–	–	+	+	††
(14)	Ags12-III	–	–	–	–	+	+	††
(15)	Ags12-V	–	–	–	–	+	+	††
(16)	Ags12-VIII	–	–	–	–	+	+	††
(17)	Ags12-XII	–	–	–	–	+	+	††
(18)	Ags12-XIII	–	–	–	–	+	+	††
(19)	Ags12-XIV	–	–	–	–	+	+	††
(20)	Ags12-XVI	–	–	–	–	+	+	††

*All samples come only from three different swine farms*.

*^+^Positive; ^†^Biofilms formation; ^†^weak biofilms; ^††^moderate biofilms; ^††††^strong biofilms; ^−^Negative*.

* *Sequences: Ags5-I (accession number: KU169148), Ags8-V (accession number: KU169146), and Ags12-II (accession number: KU169147)*.

### 16S rDNA Analyses

Analyses of 16S rDNA showed the presence of *Stenotrophomonas maltophilia* and *Acinetobacter schindleri* in eight and three samples, respectively, and *E. coli* in all the samples. The detections were as follows: *A. pleuropneumoniae* and *S. maltophilia* in some farms, and *A. pleuropneumoniae* and *A. schindleri* in others, always with *E. coli*. Additionally, other bacterial species were detected: *Provotella* spp., *Ideonella dechloratans, Novosphigobium* spp., and *Propionivibrio dicarboxylicus*, but these species never repeated between farms.

### Detection of *A. pleuropneumoniae* in Biofilms *in vitro* and *in situ*

**Figure 2:**
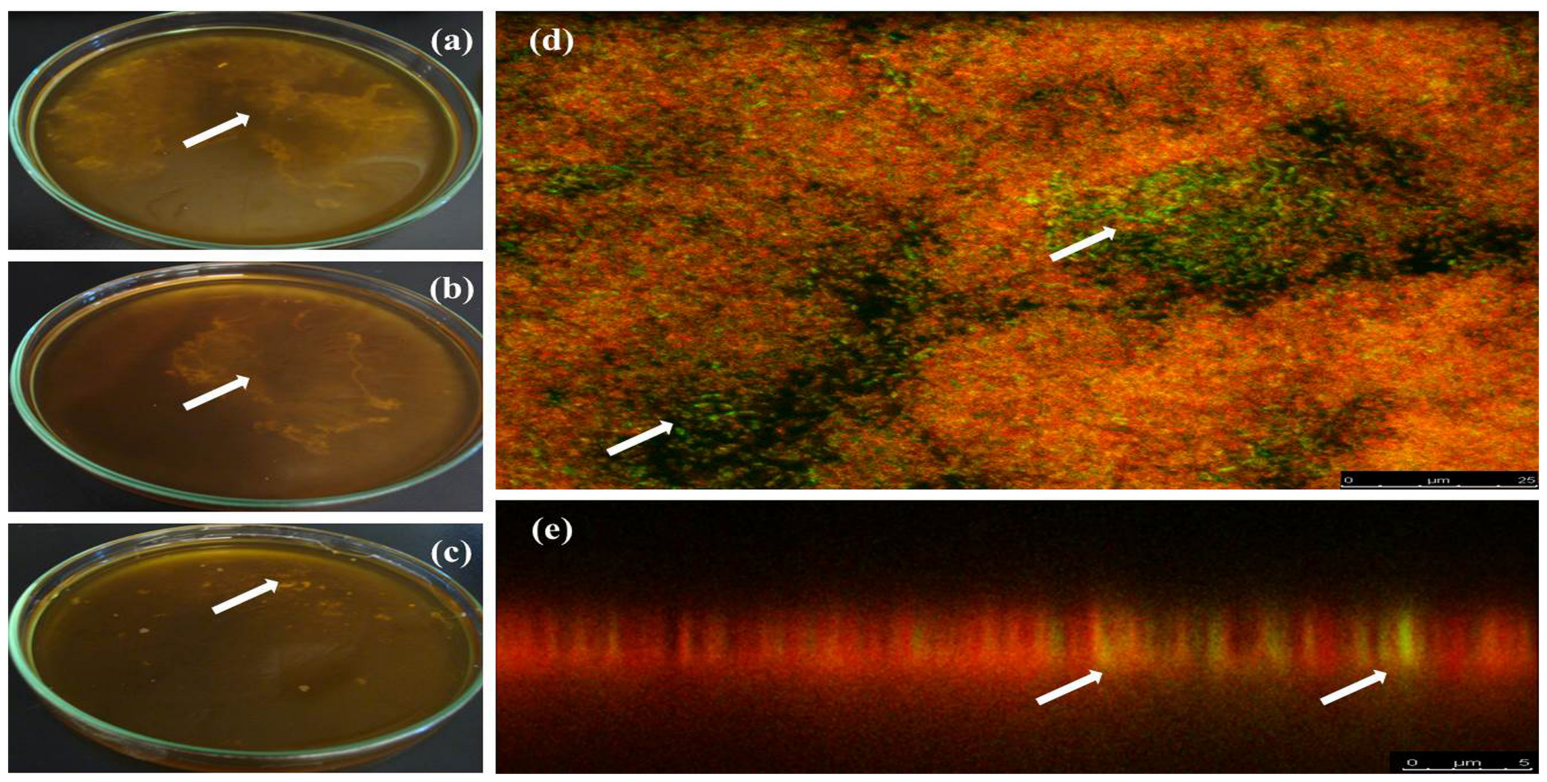
Biofilm formation by positive samples for *A. pleuropneumoniae* from drinking water. **(a–c)** Biofilms in the liquid–air interface formed *in vitro* from drinking water of one Mexican farm in three different samples (arrows show the biofilms). **(d,e)** FISH technique to detect *A. pleuropneumoniae* in biofilms from samples of drinking water from swine farms; *A. pleuropneumoniae* was detected with probes labeled with fluorescein (green) and other bacteria were labeled with ethidium bromide (red) (arrows show *A. pleuropneumoniae* label). **(a,d,e)** Ags5-I, **(b)** Ags5-II, and **(c)** Ags5-III.

**Figure 3:**
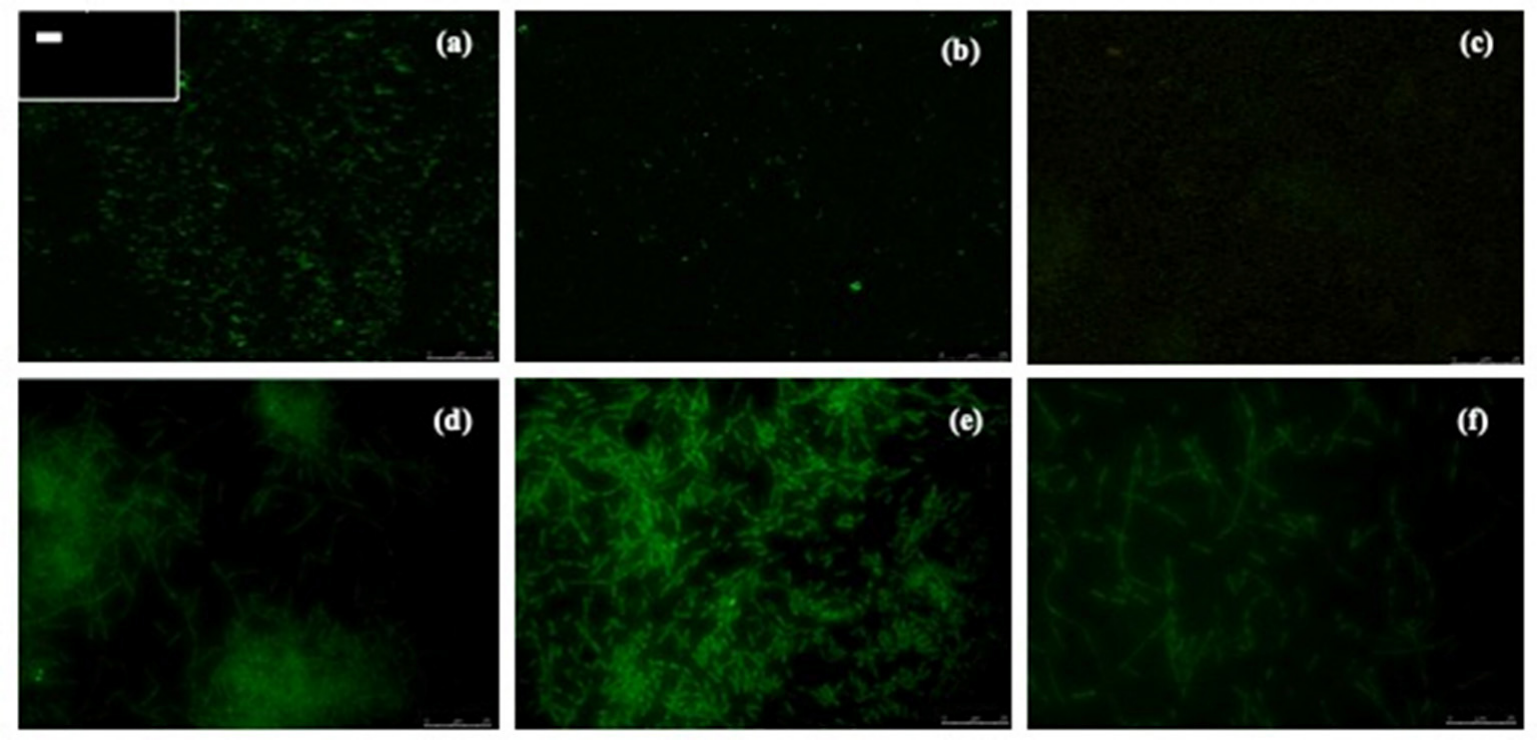
Detection of *A. pleuropneumoniae* in biofilms produced from drinking water by fluorescent *in situ* hybridization. Micrographs were taken with confocal laser scanning microscopy **(a–c)** or epifluorescence microscopy **(d–f)**. **(a)** *A. pleuropneumoniae* 1-4074 and in the box at the upper left corner (–) negative control (*E. coli* ATCC 25922). **(b,c)** *A. pleuropneumoniae* detection by CLSM. **(d–f)** *A. pleuropneumoniae* detection by EM (seen at 100×).

**Figure 4:**
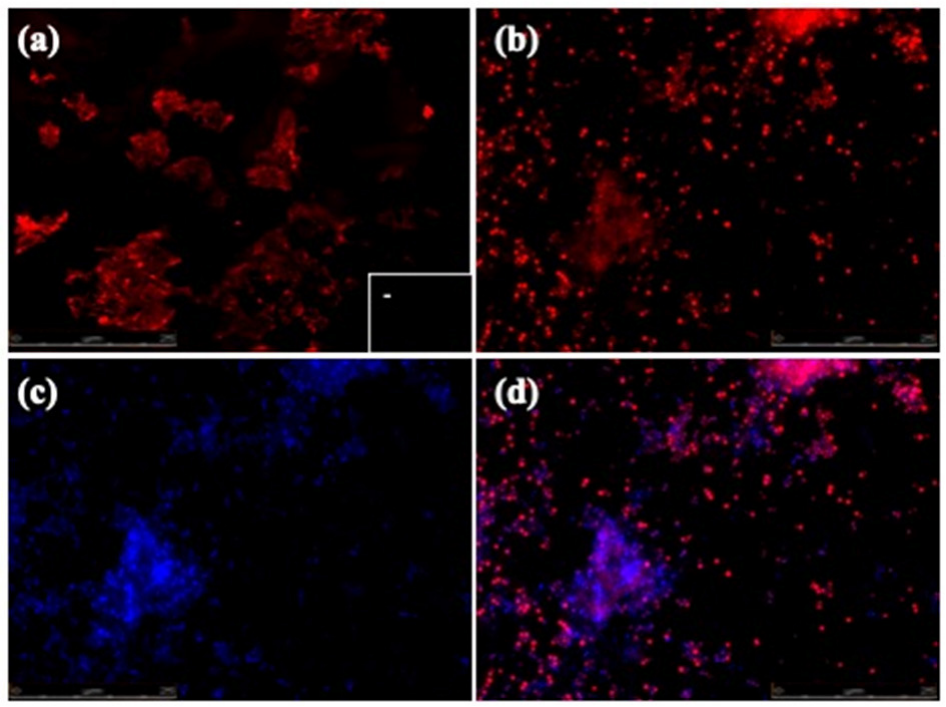
FISH of *A. pleuropneumoniae* in biofilms detected *in situ* at drinkers in swine farms. **(a–d)** Epifluorescence microscopy pictures (seen at 60×). **(a)** *A. pleuropneumoniae* 1-4074 and in the box in the lower right corner (–) specific control (*E. coli* ATCC 25922). **(b)** *A. pleuropneumoniae* positive samples from drinkers. **(c)** General DAPI staining of microorganisms in the sample. **(d)** Merge of **(b,c)**.

Biofilm formation is a strategy that allows bacteria to survive in hostile environments. Here, it was observed that the microbial communities present in water samples obtained from pig farms of Aguascalientes, Mexico had the ability to form biofilms *in vitro*. Twenty positive samples containing *A. pleuropneumoniae* were able to generate biofilm in the liquid–air interface and attached to the surface *in vitro* (Figure 2). In these samples, *A. pleuropneumoniae* was detected in biofilm by FISH (Figures 2, 3). In three samples (Ags5-I, Ags5-II, and Ags5-III), many biofilms in the liquid–air interface were observed. Besides, in these samples were detected to *A. pleuropneumoniae, E. coli*, and *A. schindleri* (Figure 2). To test if this biofilm formation was induced *in situ* in the farm environment, FISH was used directly for the slide samples obtained from drinkers at the swine farm. *A. pleuropneumoniae* was detected in 4 of 100 samples tested from biofilms obtained directly from drinkers of swine farm, together with other bacteria labeled with DAPI staining, which were not identified (Figure 4).

**Figure 5:**
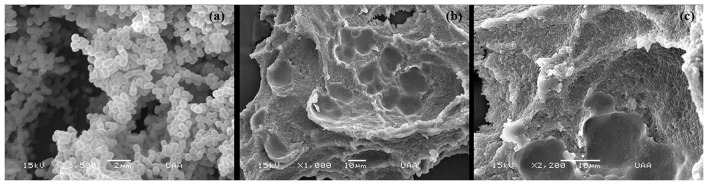
Scanning electron microscopy (SEM) of biofilms produced by positive samples to *A. pleuropneumoniae* from drinking water in swine farms. **(a)** Biofilms of *A. pleuropneumoniae* 4074. **(b,c)** Biofilms of positive samples to *A. pleuropneumoniae* from drinking water collected at swine farms.

These biofilms formed from drinking water samples of pig farms were analyzed under SEM. Although this technique does not permit the identification of *A. pleuropneumoniae* within the biofilm, it allows us to observe the structure and morphology of the biofilms. In the biofilms, the existence of bacteria in the form of bacillus and especially the production of a large amount of extracellular matrix enveloping the bacteria were observed (Figure 5).

## Discussion

The swine industry is highly affected by sanitary problems; the porcine respiratory disease complex is one of them (32). Within this complex, *A. pleuropneumoniae* is one of the main agents causing economic losses worldwide, being the causative agent of swine pleuropneumoniae (2). Álvarez et al. (33) reported in a study done in the 25 farms in the State of Yucatan, in the south of Mexico, that all sampled farms had pigs infected with *A. pleuropneumoniae*, finding serovars 1, 3, and 7. Loera-Muro et al. (34) reported a prevalence of *A. pleuropneumoniae* in 79% of the farms in central Mexico, with an incidence of 20% per farm on average. Our group has reported the presence of *A. pleuropneumoniae* in drinking water from pig farms (26, 27). In the present work, the detection of the *apxIV* gene was used as an indicator for the presence of *A. pleuropneumoniae* in biofilms from the swine drinking water, and in drinkers. This gene was reported as specific for *A. pleuropneumoniae* (28). However, although it was possible to detect *apxIV* in all samples, further testing is needed since the detection of the other toxin genes (like *apxIB* and *apxII*) was possible in a few cases. Considering this information, and that described by Rayamajhi et al. (29) we conclude that the serovar of *A. pleuropneumoniae* found in drinking water presumably belongs to serovar 7. This serovar is frequently reported in North America and Mexico (16). Moreover, as other bacteria, such as *S. maltophilia, A. schindleri*, and *E. coli*, are present in the samples, we suggest that these bacteria can supply the nutrients needed to grow in media without NAD to *A. pleuropneumoniae*. Those results suggest that multi-species biofilm formation helps *A. pleuropneumoniae* to survive in the environment of drinking water in association with other microorganisms that were detected in the samples, and probably in the biofilms (23–25). The detection of *A. pleuropneumoniae* with other bacteria in biofilms in the swine drinkers, forming multi-species biofilms, supports this observation. *S. maltophilia* is a global emerging multidrug-resistant opportunistic pathogen found in water, soil, plant rhizosphere, food, and animals (35, 36). Ryan et al. (37) reported that *S. maltophilia* can interact with the cystic fibrosis (CF) pathogen *P. aeruginosa* in multi-species biofilms. Moreover, *Acinetobacter* genus is widely distributed in nature since they are found frequently in soil, water, and dry environments. *A. schindleri*, described in 2001, could represent nonnegligible opportunistic pathogens because their routine identification is not possible by a phenotypic approach (38). Hansen et al. (39) reported that multi-species biofilms formed by *Acinetobacter* sp. and *Pseudomonas putida* promote their growth in adverse environments for their development. *P. putida* population is dependent on the benzoate excreted from *Acinetobacter* during the catabolism of benzyl alcohol, the sole carbon source. In the case of *E. coli*, Pereira et al. (40) observed that putative F pili engage typical Enteroaggregative *E. coli* (EAEC) strains in forming mixed biofilms, increasing the overall bacterial adhesion when diarrhea-isolated aggregative *Citrobacter freundii* is present. Likewise, our group found that *A. pleuropneumoniae* serovar 1 can get NAD or some of its precursors from other bacteria such as *E. coli, Streptococcus suis, Bordetella bronchiseptica, Pasteurella multocida*, and *S. aureus* when growing into a mixed biofilm (23). However, with the impossibility of achieving pure isolation of the samples, studies are necessary to confirm if these strains of *A. pleuropneumoniae* can grow without NAD supplementation due to interaction with one of these bacteria found, or the variant found could belong to serovar 7 atypically biovar II reported.

Another interesting fact is that these samples were obtained from the environment surrounding pigs, such as drinking water, suggesting that *A. pleuropneumoniae* survive in an environment biofilm. The ability of *A. pleuropneumoniae* to form biofilms has been widely demonstrated *in vitro* (10). However, there are only a few studies on the survival of *A. pleuropneumoniae* in the environment, outside the pig. Assavacheep and Rycroft (41) demonstrated that *A. pleuropneumoniae* survived only 3–4 days under controlled laboratory conditions, involving cool temperatures plus NaCl. Loera-Muro et al. (26) detected the presence of *A. pleuropneumoniae* in water from pig farms, but the *A. pleuropneumoniae* biofilm formation was not confirmed. In this study, *A. pleuropneumoniae* was detected in environmental biofilms, and it was detected in samples with other environmental bacteria, including *S. maltophilia, A. schindleri*, and *E. coli*, among others, and it was detected forming multi-species biofilms in drinkers. In nature, multi-species biofilms represent the lifestyle preferred by bacteria (21). These structures allow them to survive even in extremely adverse conditions for the development of planktonic life (19). These multi-species biofilms are regulated by a variety of inter- and intra-species interactions, which are very important for their development, composition, structure, and function (42, 43). Bridier et al. (44) evaluated the biofilm resistance of a *Bacillus subtilis* strain (ND_medical_) isolated from endoscope washer-disinfectors to peracetic acid (PAA), and its ability to protect the pathogen *S. aureus* in mixed biofilms. When grown in mixed biofilm with *S. aureus*, the ND_medical_ strain demonstrated the ability to protect the pathogen from PAA action, thus enabling its persistence in the environment. Similarly, our group found that *A. pleuropneumoniae* serovar 1 can grow and form multi-species biofilms with other bacteria belonging to porcine respiratory disease complex, like *S. suis, B. bronchiseptica*, and *P. multocida*, and with other bacteria commensal to swine (*S. aureus*) or with a human pathogen (*E. coli*) (23, 24). On the other hand, another possible explanation to the DAPI labeled is the possible presence of extracellular DNA (eDNA) in environmental biofilms where it was possible to detect *A. pleuropneumoniae*. The presence of eDNA in *A. pleuropneumoniae* biofilms was already reported by several authors (23, 45). Likewise, the presence of eDNA in environmental biofilms as part of the extracellular matrix of the same is well-reported in the literature for several bacterial species. Dominiak et al. (46) detected the highest amount of eDNA in and around the microcolonies of denitrified bacteria belonging to the genera *Curvibacter, Thauera*, the ammonium-oxidizing *Nitrosomonas*, and the nitrite-oxidizing *Nitrospira*. Tang et al. (47) quantified eDNA over time during planktonic growth and biofilm formation in the strain *Reinheimera* sp. F8 and in three other environmental isolates belonging to the genera *Pseudomonas, Microbacterium*, and *Serratia*. They observed that eDNA was important for the initial attachment in all strains, and DNase treatment reduced biofilm formation in three of four strains. Hatrhoubi et al. (45) and Loera-Muro et al. (23) observed changes in the composition of the biofilm structure of *A. pleuropneumoniae*. These changes were mainly in the composition of eDNA from the extracellular matrix, which has structural functions when the biofilm was subjected to some environmental stress (presence of antibiotics or absence of nutrients).

Finally, in this study, *A. pleuropneumoniae* biofilms were detected in pig drinkers, and these may represent a continual inoculum for animals, assuming that it comes from the infected pigs that use the drinkers. For example, the pathogen *Campylobacter jejuni* can form biofilms in the water supplies, and plumbing systems of animal husbandry facilities, where biofilm may provide a continual inoculum for domesticated animals (48, 49). However, more studies are needed to confirm whether the presence of *A. pleuropneumoniae* in biofilms in drinkers could represent a source of inoculum for animals, as is the route of transmission through direct contact between pigs (2).

In conclusion, our data suggest that *A. pleuropneumoniae* form environmental biofilms in samples from drinking water, and in drinkers at swine farms. In addition, the detection of biofilm formation with other different bacteria, such as *S. maltophilia, A. schindleri*, and *E. coli*, showed that *A. pleuropneumoniae* can survive in the environment in multi-species biofilms.

## Data Availability Statement

The datasets presented in this study can be found in online repositories. The sequences are deposited in the GenBank: Ags5-I (accession number: KU169148), Ags8-V (accession number: KU169146), and Ags12-II (accession number: KU169147).

## Author Contributions

AL-M developed the hypothesis, designed and performed all the experiments, and contributed to the manuscript. FR-C, AM-F, and EM contributed to the research plan and experiments. AL-M and AG-B contributed to the experimental design and manuscript preparation. FA-G and AG-B contributed to the conception of the idea, the experimental design and manuscript preparation, and the funds to carry out the research. All authors contributed to the article and approved the submitted version.

## Funding

This project was supported by a grant from Universidad Autonoma de Aguascalientes, Mexico Award number: PIBT16-3, CONACYT, Mexico (No. 258863), and Special Resource UAA for Research 2017.

## Conflict of Interest

The authors declare that the research was conducted in the absence of any commercial or financial relationships that could be construed as a potential conflict of interest.

## Publisher's Note

All claims expressed in this article are solely those of the authors and do not necessarily represent those of their affiliated organizations, or those of the publisher, the editors and the reviewers. Any product that may be evaluated in this article, or claim that may be made by its manufacturer, is not guaranteed or endorsed by the publisher.
