# Supplementary material for: Actinobacillus pleuropneumoniae Surviving on Environmental Multi-Species Biofilms in Swine Farms
Source: Front Vet Sci. 2021 Sep 30;8:722683. doi: 10.3389/fvets.2021.722683 (PMC8515031; doi:10.3389/fvets.2021.722683)
Supplement: Supplementary file 1 [file Data_Sheet_1.docx]

Supplementary Material

*Actinobacillus pleuropneumoniae*surviving on environmental multi-species biofilms in swine farms

Abraham Loera-Muro^1^, Flor Y. Ramírez-Castillo^2^, Adriana C. Moreno-Flores^2^, Eduardo M. Martin^2^, Francisco J. Avelar-González^3^, and Alma L. Guerrero-Barrera^2^

*** Correspondence:** Ph.D Alma Lilián Guerrero Barrera: alguerre@correo.uaa.mx

# Supplementary Figures and Tables

## Supplementary Figures


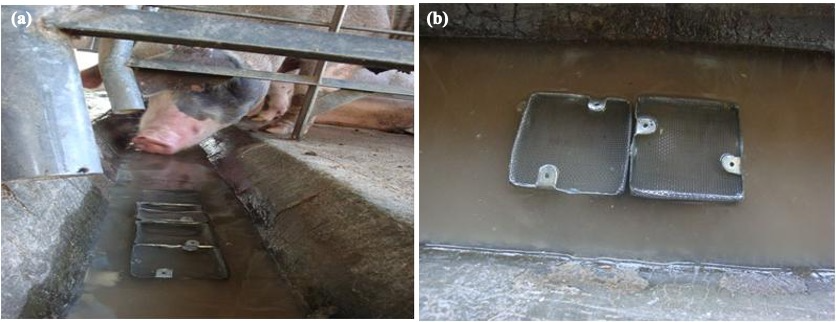


**Supplementary Fig. S1.** (a and b)Watering places where water samples were obtained and where the portable device to biofilms formation assays were placed directly at the swine farm. Also, a first prototype of the portable device for obtaining biofilms *in situ* is observed.

**Supplementary Fig. S2.** Detection by PCR of *apx* toxin genes. (a) *apxIV* gene. Lane 1 – DNA ladder (Fermentas); Lane 2 *–A. pleuropneumoniae* serovar1-4074; Lane 3 –*E. coli* ATCC 25922; Lane 4 to 23 – PCR of DNA extracted from drinking water samples using a pair of primers versus *A. pleuropneumoniaeapxIV* gene. (b) *apxIB* gene. Lane 1 – DNA ladder (Fermentas); Lane 2 – *A. pleuropneumoniae* serovar 1-4074; Lane 3 – *A. pleuropneumoniae* serovar10; Lane 4 – *A. pleuropneumoniae* serovar3; Lane 5 to 8 – PCR of DNA extracted from drinking water samples using a pair of primers versus A. pleuropneumoniae *apxIB* gene. (c)*apxII* gene.Lane 1 – DNA ladder (Fermentas); Lane 2 – H*2*O; Lane 3 – *A. pleuropneumoniae* serovar 10; Lane 4 – *A. pleuropneumoniae* serovar1-4074; Lane 5 to 6 – PCR of DNA extracted from drinking water samples using a pair of primers versus A. pleuropneumoniae *apxII* gene.
